# Development and Successful Application of a Tree Movement Energy Harvesting Device, to Power a Wireless Sensor Node

**DOI:** 10.3390/s120912110

**Published:** 2012-09-04

**Authors:** Scott McGarry, Chris Knight

**Affiliations:** Commonwealth Scientific and Industrial Research Organisation (CSIRO), P.O. Box 330, Newcastle, NSW 2300, Australia; E-Mail: scott.mcgarry@csiro.au

**Keywords:** energy harvesting, energy scavenging, tree movement, tree sway, tree energy, sensor network, sensor node, wind energy

## Abstract

Wireless sensor networks are becoming increasingly more common as a means to sense, measure, record and transmit data for scientific and engineering evaluation, remotely and autonomously. Usually, remotely located sensor nodes are powered by batteries which are recharged by solar or wind energy harvesters. Sometimes nodes are located in areas where these forms of energy harvesting are not possible due to local conditions, such as under the canopy of a forest. This article outlines the design and testing of a device capable of harvesting energy from tree movement, and shows the device powering a wireless sensor node continuously. The device uses the force and displacement of the movement of a tree trunk (of a 6 m tall tree) to drive an electromagnetic generator that recharges a nickel metal hydride battery. The battery stores the energy from which a ∼0.5 mW wireless sensor node is powered continuously. This demonstrated method of energy harvesting may allow the placement and powering of nodes in locations previously not possible.

## 1. Introduction

### 1.1. Background

The authors have previously conducted experiments which determined that the movement of a tree would be able to provide enough energy to power a wireless sensor node [1]. A number of approaches were investigated, including “inertial” and “tethered” energy harvesting, of which only the tethered approach was considered feasible. This work was performed to provide a possible energy harvesting solution for sensor nodes and networks, for example [2], located below forest canopies, where other forms of energy supply [3] were not considered feasible. The research [4,5] showed evidence that suggested that under some forest canopies there is not enough wind or sunlight to allow the use of photovoltaic or wind energy harvesters to power wireless nodes. The results of this work suggested that the level of energy available through use of a “tethered” type tree movement energy harvesting device, showed promise as an energy source for a wireless sensor node.

Also listed in [1] were a number of patents taken out in the USA between 2004 and 2011 [6–9], for devices supposedly capable of harvesting energy from the movement of trees. From the work carried out in [1], the authors determined that the power claims made by a number of the patents seemed excessive. It is not apparent that any device had been constructed, before (or since) the construction of the experimental prototype, outlined in this article, and that the claims made cannot be verified or proven, as no other scientific study of the movement energy of trees has been published. Of those patents that exist, Kim *et al.* [7] describes a device that operates via the “inertial” approach and was therefore believed to be infeasible. The others describe devices which use the “tethered” approach. Of those devices using the tethered approach, the authors believe that shortcomings exist for each.

Mooring [6] describes a device that is designed to be located at ground level, attached to a tree via a “*cable tether…attached to the main tree trunk approximately 2/3 of the tree's height*.” The device uses a spring loaded arm that operates a ratchet mechanism, to progressively wind a spring motor until a particular level of elastically stored energy is reached, whereupon the energy is released to drive an electrical generator with flywheel. The authors believe there is an inherent disadvantage with this device; the use of springs to both return the arm to the neutral, or starting, position and to store energy before generation. These springs add to the stiffness of the tree energy harvester system, thus reducing the overall movement of the tree and energy harvester. The magnitude of the energy reduction is a function of the overall spring constant, but the authors believe that without careful design and selection of the tether, can be great enough to overwhelm the movement of the tree. No indication as to the importance of tether selection to avoid movement absorbing sag and elasticity, nor the arrangement of the tether, nor the affects these choices have on the required spring stiffness, is given in the patent.

The patent by Rees and Faigen [9] describes a distributed system of many tree energy harvesters they refer to as “*Pull Retract Generators (PRGs)*”, located between trees, with tethers connected from one tree to another, at an angle the authors can only assume is horizontal. The PRG also suffers from the use of a spring to keep the tether taut, the effects of which are exacerbated in this application if the tethers are arranged horizontally. This is due to maximised, movement absorbing, sag from the self weight of a horizontally arranged tether. The increased spring rate required to overcome sag in the tether, acts to reduce the relative movement between trees. This patent does refer to the optimal design of installation being a complex process, and quotes “*The optimal placement of the PRGs (e.g., at what height they should be hung and between which trees they should be suspended)….must be determined empirically*”. A further perceived disadvantage of the PRG is the use of many gear meshes between the tether and the generator, adding friction, and therefore force, required to start and move the generator.

It has been pointed out to the authors that the concept of harvesting tree movement energy existed as early as 1981, when the movie “E.T.” was produced. The main character, E.T., used the harvester to power a device, which he/she used to “phone home”. This predates all other known work.

### 1.2. Work Undertaken

The authors hypothesised that it was possible to both predict the movement energy available for extraction, and to build a device to harvest the energy contained in the movement of a tree to power an existing wireless sensor node. To this end, an experimental prototype tree movement harvesting device, operating via the “tethered” approach, was conceived, designed, built and tested, to harvest energy from a 6 metre tall eucalypt tree, typical of many trees found in native Australian forests.

Before construction of the device, a free movement analysis of the tree, selected for use in experimentation, was performed over a 69.5 hour period. This was done to determine the magnitude of movement energy in the tree, and how much of that energy could be harvested by a device operating via the principal we devised.

**Figure 1.**
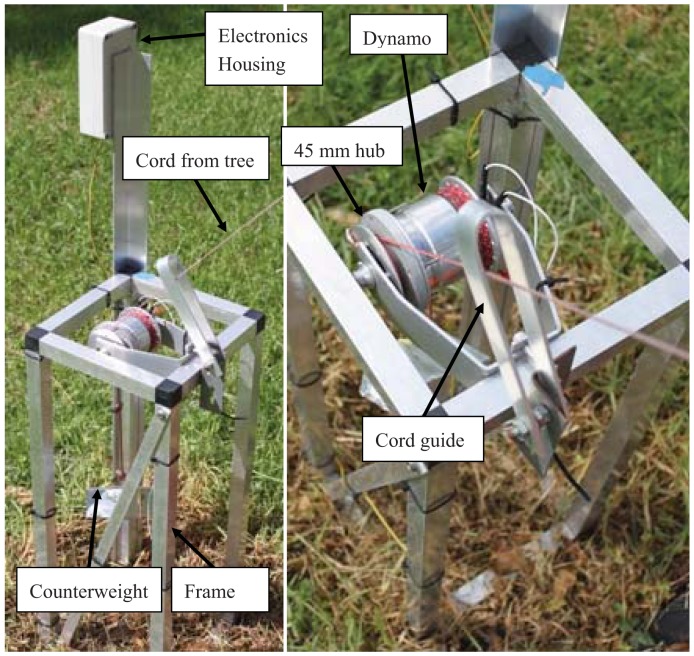
Tree movement energy harvesting device.

The device consisted of an electromagnetic generator or dynamo, mounted in a frame, located on the ground a number of meters from the host tree. The dynamo operated rotationally and was driven by a cord wrapped around a hub on the dynamo. The other end of the cord was attached to the trunk of the host tree which pulled the cord as the tree swayed away from the device, thus driving the dynamo and generating electrical energy. To keep the cord taut during tree movement toward the device, a counter weight was attached to the dynamo by another cord, which applied a torque in the opposite direction to that of the cord attached to the tree (see Figure 1).

Analyses of various characteristics of the device were performed, the device was modified, tests were carried out and improvements gradually made, with the aim of enabling the device to power a wireless sensor node continuously. The AC electrical output of the dynamo was connected to an electrical circuit, which rectified the charge to DC, for storage of the energy and power to a wireless sensor node. One of two prototype harvesting devices constructed powered a wireless sensor node continuously for almost 1 year, when the experiment was terminated.

## 2. Methodology

### 2.1. Sensor Node and Network

The aim of the work outlined in this article was to design and build a device capable of harvesting tree movement energy to power a wireless sensor node. The tree powered sensor node (a Fleck3B™) was programmed to be part of an existing sensor network comprised of similar devices that included a number of transmitting nodes and one receiving node, similar to networks described in [10,11]. The tree powered node was a transmitting node, sending data including on board temperature, supply voltage, and sample number from the time of power up. This node was programmed to sample and transmit data once every 15 minutes, when powered continuously. The information from this and other nodes was transmitted to a receiving node and stored on a DataTaker DT80 data logger [12], connected via a serial communication port.

Another of the nodes in the network was connected to a Delta Ohm HD2003, 3-axis ultrasonic anemometer [13]. This node was also programmed to sample and transmit various data once every 15 minutes, and included wind speed in three axes. Again, this data was transmitted to the receiving node and recorded on the data logger.

### 2.2. Selection of the Tree Used for Experimentation

Before design and construction of the tree movement harvesting device began, a tree was selected for use during experimentation and development using a number of criteria. The criteria included that the tree be:

- easily accessible to researchers,
- secure from vandalism/theft, so that hardware could remain attached over extended periods of time,
- surrounded by other trees to best replicate the conditions in a forest,
- small (short) enough that significant displacement would occur within reach from ground level with use of tools,
- sufficiently healthy and foliaged, to provide force for movement during wind events.

**Figure 2.**
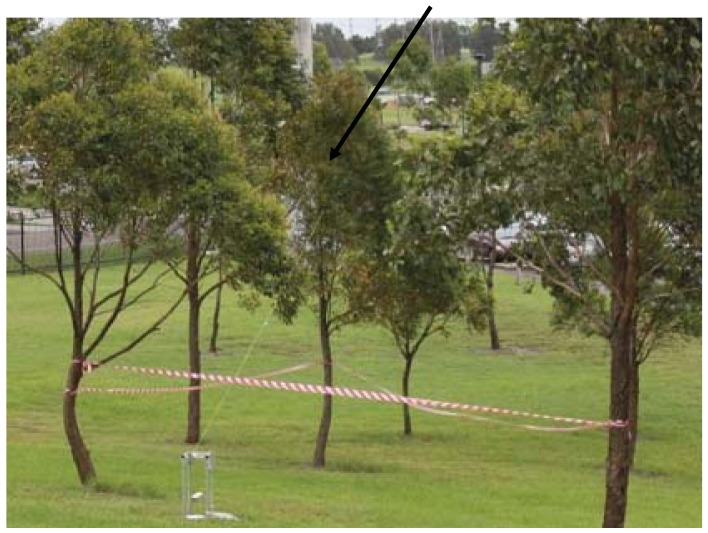
Tree used for experimentation and development of movement harvesting device, as indicated by arrow.

The tree chosen for use throughout the experiments conducted was in a location within the grounds of CSIRO Energy Centre, at Newcastle, Australia, latitude 32°53′04.5″S, longitude 151°43′39.75″E. At the beginning of experimentation and development, the tree was approximately 6 m tall, with a base circumference of 0.46 m. The tree was tentatively identified as a *Eucalyptus microcorys* species (voucher specimen CANB 803880, Australian National Herbarium). It was located on a gentle slope surrounded by a handful of other trees of a similar to slightly larger size. See Figure 2.

### 2.3. Pre Development Analysis

Initial experimentation performed upon the tree described in Section 2.2, included selecting a point on the trunk, 4 m above ground level, at which the elastic stiffness of the tree was measured. The stiffness was measured at an angle to horizontal of 11 degrees, by tying one end of a cord to the point on the tree trunk, and attaching the other end to a calibrated spring balance. During a calm period, force was manually applied to the free end of the balance, and the movement of the other end of the balance measured with a tape measure. The elastic stiffness of the tree was determined to be close to 400 N/m, using a 100 N spring balance deflected by approximately 250 mm; a more accurate figure was difficult to determine as there is virtually always some wind present and some small movement occurring. However, analytical methods used in [1] to determine the elastic stiffness of tree trunks were used to verify the value of 400 N/m as being a representative figure.

Further experimentation was conducted to determine the “free” movement of the tree in wind, for a period of about 3 days. A draw wire potentiometer [14] was connected to the trunk, and the chassis of the potentiometer pegged to the ground, resulting in a 17 degree angle between the cord and horizontal. The movement of the trunk was then determined at a sample rate of 10 Hz, for a period of 69.5 hours. The potentiometer incorporated a weak spring to keep the cord taut, resulting in a load applied to the tree trunk of less than 1 N.

Analysis of the data determined base characteristics for the tree movement energy harvesting device. The analysis was based on the assumption that the energy harvesting device would use an electromagnetic transducer to convert the movement to electrical energy. It was further assumed that the device would offer a constant resistance force (drag) to the actuation movement by the tree, due to the electro-magnetic energy conversion process. Based on these assumptions, the following process was followed to determine the optimum electromagnetic drag for maximum energy harvesting:

Each local minimum (lower turning point) in the data was found, indicating where the tree movement changed from negative to positive. Each subsequent local maximum (upper turning point) was located and the change in displacement (Δ*x*) between each lower and upper point calculated. Each positive change in displacement of free movement was converted into a change in applied force by the tree (Δ*F*), based on the elasticity of the tree, using:

(1) ΔF=kΔx

where *k* = 400 N/m.

Convert each change in displacement into a change in elastic energy in the tree (Δ*E_tree_*) using

(2) $$\Delta E_{\text{tree}}=\overset{1}{\;}/\underset{2}{\;}\Delta F\Delta x=\overset{1}{\;}/\underset{2}{\;}k{\left(\Delta x\right)}^{2}$$

The sum of these figures gives the total amount of elastic energy in the trunk,

(3) $$E_{\text{tree}}=\sum \Delta E_{\text{tree}}$$

Given that the harvester described in this article is only extracting energy above a certain force or electromagnetic drag threshold (*F_threshold_*), for each change in force greater than the threshold, the extractable energy (*ΔE_extractable_*) was found and totalled. This can be calculated for each Δ*F* using:

(4) $$\Delta E_{\text{extractable}}=\Delta s\times F_{\text{threshold}}$$

where Δ*s* = net displacement:

(5) $$\Delta s=\max(0,\frac{\Delta F-F_{\text{threshold}}}{k})$$

Therefore:

(6) $$\Delta E_{\text{extractable}}=\max(0,(F_{\text{threshold}}\times (\frac{\Delta F-F_{\text{threshold}}}{k}))$$

**Figure 3.**
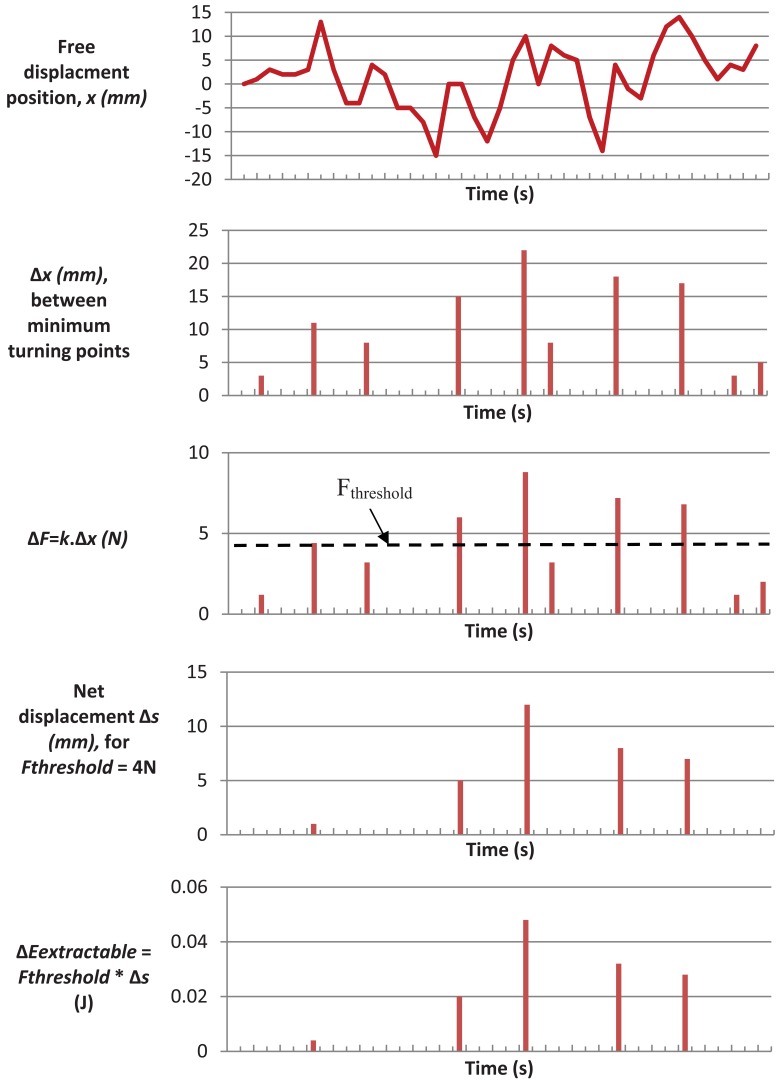
Example transformation from free displacement to extractable energy.

A plotted example of the above process is given in Figure 3, using an arbitrary free displacement plot and value for *F_threshold_* = 4 N.

The sum of all Δ*E_extractable_* (ΣΔ*E_extractable_* = *E_extractable_*) were then found for any given value *F_threshold_*. Various values of *F_threshold_* were then tried iteratively, starting at *F_threshold_* = 0.01 N and incrementing by 0.01 N each pass, to maximise *E_extractable_* and find the optimum value of *F_threshold_*. The final figure of *E_extractable_* was compared to the value of *E_tree_*. Results of this analysis are summarised in Section 3.1.

This analysis was considered to be a useful starting point for design and development of the tree movement energy harvester. It was known that the final tree harvesting device would not have a constant electromagnetic drag, but that at low speeds it was assumed approximately constant and thus a useful method.

### 2.4. Development and Design Trade Offs

To build the experimental tree movement energy harvesting device, it was decided to keep the operating principle simple, reliable and as tuneable as possible. To this end it was decided to use a rotating generator, operated by a cord wrapped around a hub connected to the generator, and actuated by the tree (as described in Section 1.2). A counter weight attached to a second cord would keep the tree cord taut as the tree moved toward the device. Further, an electromagnetic transducer was deemed the best option, due to the mature state of the technology, the availability of performance data and the ability to obtain such generators easily. Such generators exist specifically to work outdoors and in harsh environments, in the form of bicycle dynamos. Figure 1 shows the arrangement of the dynamo, hub, cords and counterweight, in the final form.

**Figure 4.**
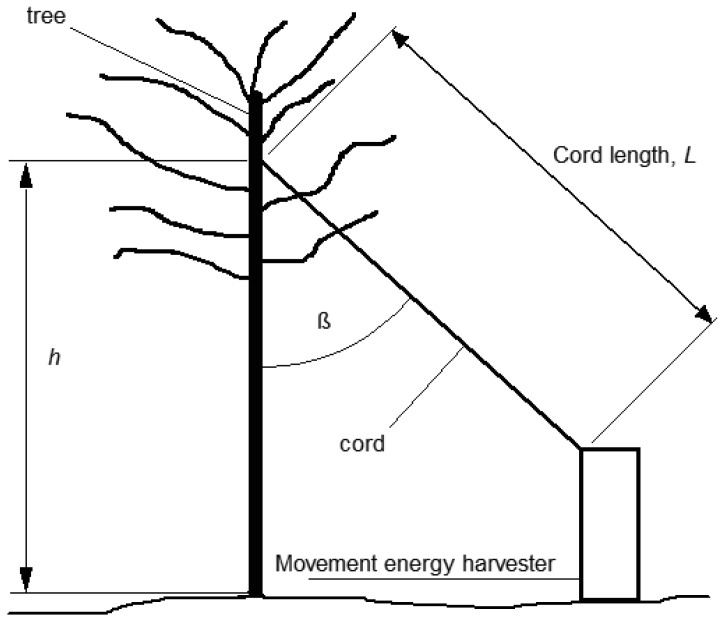
Side view sketch of tree and movement energy harvesting device.

The mass of the counterweight was sized such that at the diameter around which its cord was wrapped, the force (weight) due to gravity applied a torque slightly greater than the drag torque of the generator. The arrangement of the device in relation to the tree to which it is connected is shown in Figure 4. The relationships and trade-offs between the arrangement of the energy harvesting device, and the characteristics of the device, affected the overall performance.

Aims of cord selection and arrangement:

- reduce cord sag by minimising the mass of the cord, by decreasing thickness, light weight material selection, or by minimising angle ß of the cord;
- reduce cord sag by minimising mass increase due to water absorption of the cord, through careful material selection;
- reduce elasticity of the cord by maximising the tensile stiffness of the cord through increasing thickness or careful material selection;
- reduce elasticity of the cord by minimising the cord length, by minimising angle β of the cord.

It is obvious that some design trade-offs are required due to competing characteristics e.g., thin light weight cord to reduce sag *versus* thicker tensionally stiff cord to reduce elasticity. It should also be obvious that the angle β of the cord to the tree was an important parameter in maximising the output, however a limiting factor was that as the angle was reduced the optimum drag for the device increased due to the trigonometric relationship between the movement of the tree and the movement of the device. Drag could only be increased based on the diameter of the hub; unless a secondary gear system was employed, adding complexity and possibly some unwanted slack in the system. As such, drag was limited to an upper value (approximately 10 N static), based on the smallest hub diameter that fitted the dynamo, thus limiting angle β to a minimum best performing value.

A further complicating factor to the issue of cord and cord angle selection was the influence of the connection point of the cord within the tree *i.e.*, the height *h*. As *h* is increased it affects cord length and/or cord angle. It was also hypothesised that it would also affect the optimum drag for any given wind event, as it has been observed that tree displacement in a wind is greater nearer the top, and that trunk elasticity reduces in magnitude, the higher up the tree it is measured.

### 2.5. Characterisations and Descriptions of Components

#### 2.5.1. Electromagnetic Generator (Dynamo)

A number of possible mechanisms to allow the tree to drive a generator were considered before the final design was arrived at. It was decided early within the design process that an off-the-shelf electromagnetic generator was to be used, primarily to reduce the number and complexity of components requiring individual design and construction. A small generator was sought, with the knowledge that if an average power output of milliwatts was required, a peak output a number of orders of magnitude greater than this would probably be necessary due to the intermittent behaviour of tree movement. It was decided that a dynamo designed for use on bicycles would not only be powerful enough, but efficient, reliable and capable of withstanding life outdoors. Further, it produced a voltage within the range of that used by the wireless sensor node to be powered. The technical details of the dynamo chosen, a Schmidt Nabendynamo SON28, are outlined in Table 1.

**Figure 5.**
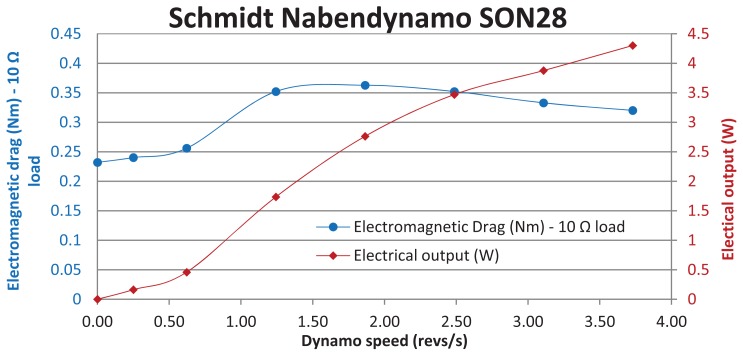
Characterisation of Schmidt Nabendynamo SON28, 10 Ω fixed load.

Testing indicated that the electromagnetic drag offered by the dynamo is not constant over its entire designed speed range, as shown in Figure 5. Results indicate that a majority of tree motion produces a dynamo rotational speed of less than 1 rev/s.

#### 2.5.2. Hub

The hub chosen for the device was 45 mm in diameter. This proved useful in combination with the other parameters of the device. This was the smallest hub that could easily be fitted to the dynamo, without modification to the dynamo, and without designing and implementing a secondary drive mechanism to further magnify the dynamo rotation to cord displacement ratio.

#### 2.5.3. Cord

After much experimentation with cords ranging from 3 mm nylon boating cord (too heavy, and water absorbent) to high strength fishing line (too elastic), to fibreglass tow (too much friction from intertwining when wrapped over itself) the cord used in the final arrangement was made from Dyneema™ as specified in Table 1.

#### 2.5.4. Counterweight (Suspended Mass)

The counterweight used in the final arrangement was 770 g, with the 3 mm thick nylon attachment cord wrapped around the dynamo body (diameter 62 mm). This arrangement provided a constant torque to the dynamo of 0.245 Nm, slightly greater than the start up drag torque of the dynamo. Heavier weights were used for experimentation, primarily to overcome slack or sag in early cord arrangements (see Section 2.5.3), but were unnecessary for the final cord selection.

#### 2.5.5. Wireless Sensor Node, Energy Harvesting Circuit and Battery

**Figure 6.**
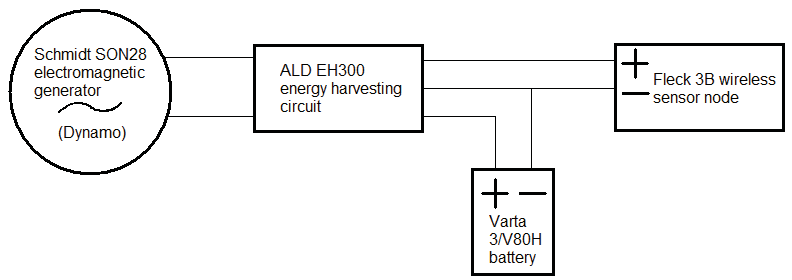
Electrical schematic of generator, energy harvesting circuit, battery and wireless sensor node.

The logic arrangement of the components within the electric housing is shown in Figure 6. For simplicity, an ALD EH300 energy harvesting circuit was used. The ALD EH300 provided rectification and allowed AC electrical energy to be captured and stored from input voltages lower than the battery voltage. This provided a convenient means of capturing the energy, although no direct comparison to other possible circuits or devices was performed. See for Table 1 outlining technical data of the hardware.

#### 2.5.6. General Arrangement

The height at which the device was connected within the tree (*h* = 5.0 m) was the highest point of those used for experimentation. The resultant angle was a function of the distance from the movement energy harvester to the tree (β = 37 degrees). Little experimentation went in to changing this distance, as hub diameter was easier to alter to account for the change in ratio between tree movement and dynamo rotation. The cord length (*L*) resulted at 5.3 m.

The orientation of the cord relative to the True North was at an azimuth of 112.5 degrees, from the energy harvesting device to the tree. This angle was dependent on the predominant wind direction for the site selected and on access to the trunk through gaps in foliage for the actuating cord. The cord was attached approximately parallel to the predominant wind direction.

### 2.6. Analysis of Device Performance

The analysis of the device performance primarily involved recording and observing the battery charge of the sensor node, powered by the movement harvesting device, across the period of operation. This data was compared to wind velocity data. Total energy consumed by the wireless sensor node was calculated, based on known consumption, and compared to the figure of *E_extractable_*, calculated in the free movement analysis. A lower bound estimate for system efficiency was made.

## 3. Results and Discussion

### 3.1. Pre Development Analysis of Free Tree Movement

**Figure 7.**
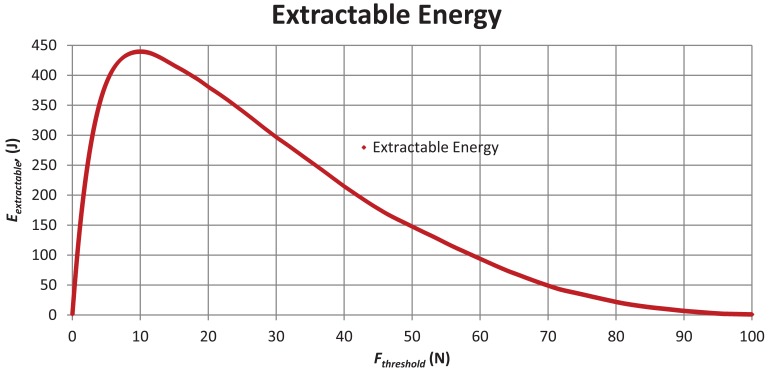
Extractable energy (*E_extractable_*) *versus* resistance force threshold (*F_threshold_*).

The optimum resistance threshold (*F_threshold_*) value for the energy harvesting device was calculated to be 10.0 N, at a connection point at the arbitrarily selected height of 4.0 m (where *k* = 400 N/m), from data plotted in Figure 7.

Calculations showed the total value of movement energy of the tree (*E_tree_*), during the ∼3 day experiment, to be 714.1 J. A figure of 439.7 J was calculated to be extractable by the fixed mechanical resistance harvesting device (*E_extractable_*), or 152 J per day, which compares favourably to the electrical energy required to power the Fleck3B wireless sensor node for the same period (∼125 J, at ∼45 J per day). The average wind speed during the three day free movement experiment was 2.40 m/s, with a recorded peak of 7.5 m/s.

### 3.2. Performance of the Device

In its final state, the electromagnetic force threshold, at the tree was 12.3 N, in the horizontal direction, at the 5.0 m connection point (which provided the most energy of various positions tested [19]). This was calculated from the 20.4 N resistance at the harvesting device, of which half was electromagnetic drag and the rest due to gravity acting on the counterweight, acting at an angle of ß = 37 degrees.

**Figure 8.**
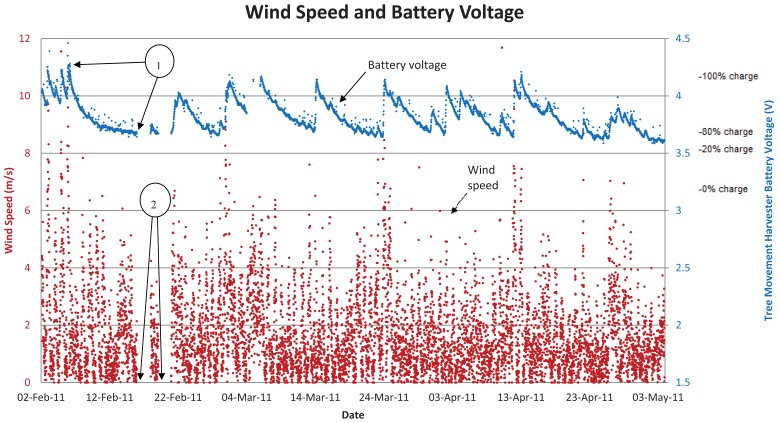
Tree powered sensor node battery voltage and wind speed; representative sample, February to May 2011.

The average wind speed for the period of testing (23 December 2010 to 23 November 2011) was 2.50 m/s, with recorded peaks as high as 15.0 m/s. Figure 8 shows the battery voltage of the sensor node powered by the tree movement energy harvester *versus* the wind velocity at the site, for a representative period of operation, early February 2011 to early May 2011. The complete deployment ran from December 2010 to November 2011. Of note, balloon 1 on Figure 8 shows where the counterweight detached during strong winds, on 6 February, with reattachment not occurring until 16 February. Balloon 2 indicates where the data logging node failed intermittently.

Over the full period of operation the harvester provided an average of more than 45 J per day to keep the node running. The amount of energy extracted by the device from the tree (*E_extractable_*) was, therefore, significantly larger than this amount, due to the inefficiencies of the system. These inefficiencies included those of the dynamo itself (<65% efficient), the battery charging/recharging process, the ALD EH300 energy harvesting circuit, and of the node itself due to supply energy, at voltages higher than those required, being wasted by the on-board voltage regulator. Assuming the system was extracting no more than 152 J per day, the calculated maximum value of extracted energy from the free movement analysis, the total system efficiency was at least 30%. The authors believe this is a good lower bound estimate of system efficiency, but that the actual figure was greater than this. This implies that less than 152 J per day of movement energy was extracted, and that the tree energy harvester is, therefore, not optimised.

## 4. Discussion

The aim of the experiment, to power a wireless sensor node through harvesting tree movement energy, was achieved, and for a significant length of time. The wireless sensor node was powered continuously for the first 10 months of deployment, and intermittently for more than another month before battery failure. This compares to an expected battery life of ∼21 days without any form of recharging. The success of this experiment gives promise to the successful application of such technology in a location where sub-canopy solar and wind resources don't sufficiently exist. However, it is expected that this may, perhaps, be a slightly more difficult task, due to the wind shading of trees by other trees. This question remains to be answered.

The calculated optimum value of the electromagnetic drag, (*F_threshold_*) of a theoretical tree movement energy harvester of 10 N was quite similar to the final value arrived at via experimentation (of 12 N), although the final connection point was at higher point on the tree trunk (5.0 m). As such this method was considered a valuable first step in determining a value of the mechanical resistance of a harvester, and the extractable movement energy within a tree, with the aim of designing a tree movement energy harvester. Useful future research would include determination of an optimum point on a tree trunk from which to harvest energy.

As can be seen from viewing the tree node sensor data plots, there are a number of gaps in the data. These were due to various problems within the wireless sensor network itself, including battery failure of the data logger at the receiving node and intermittent transmission failures within the network. Additionally, there were a number of failures at the wind velocity sensing node. The process of harvesting energy from a tree and using that energy to run a wireless node has proven to be remarkably stable.

A particular improvement to the current tree movement harvesting device has been envisaged, to allow a larger proportion of the available energy (*E_tree_*) to be harvested; a variable diameter hub. That is, a hub which varies in diameter as a function of the position of the tree. This could be done in the form of a spiral around which the cord is wrapped. Small movements, and therefore small applied forces below the existing threshold drag of the tree, could rotate the dynamo near the equilibrium position through use of a large driving radius at the hub. Larger movements (and forces) of the tree would necessitate a smaller radius to overcome the drag, thus providing greater rotation of the dynamo.

## 5. Conclusions

Harvesting the energy of tree movement to power a wireless sensor node is possible, and has been achieved. The next logical step is to apply this principal to a practical situation where power is required for a sensor node, and where sub canopy wind and solar resources are not sufficient to provide the required energy. The challenge is to harvest the required amount of energy such a situation, where the available trees are within a dense forest rather than a sparsely vegetated garden setting.

## Figures and Tables

**Table 1.** Tree Movement Energy Harvester Technical Information.

**Device**	**Manufacturer**	**Model**	**Technical Details**
Generator (Dynamo)	Schmidt	Nabendynamo SON28 [15]	Rated power: 3 Watts, see Figure 5 Rated voltage: 6 Volts AC Maximum quoted efficiency (mechanical to electrical): 65%
Hub	CSIRO	-	Diameter: 45mm
Cord	Picasso	Dyneema™	Material: Dyneema™ [16] (Ultra high molecular weight polyethylene) Yield strength: 1.7 GPa Young's Modulus: ∼70 GPa Diameter: 1.5 mm Mass per unit length: 1.49 g/m
Counter-weight	CSIRO	-	Mass: 770 g Dynamo diameter: 62 mm
Wireless Sensor Node	CSIRO	Fleck 3B™	Supply Voltage: 3.2 V to 8 V Average current draw: 145 μA (as programmed) Average power consumption: 535 μW (@ 3.7 V, as programmed)
Battery	Varta	3/V80H [17]	Nominal voltage: 3.6 V Nominal capacity: 70 mAh Chemistry: Nickel Metal Hydride
Energy harvesting circuit	ALD	EH300 [18]	Voltage input range: 0 V to 500 V, with optimum performance over 4 V Voltage output range: 1.8 V to 3.6 V, with the ability to directly pass a >3.6 V input.
